# Adult and iPS-derived non-parenchymal cells regulate liver organoid development through differential modulation of Wnt and TGF-β

**DOI:** 10.1186/s13287-019-1367-x

**Published:** 2019-08-15

**Authors:** Ernesto Goulart, Luiz Carlos de Caires-Junior, Kayque Alves Telles-Silva, Bruno Henrique Silva Araujo, Gerson S. Kobayashi, Camila Manso Musso, Amanda Faria Assoni, Danyllo Oliveira, Elia Caldini, Jonathan A. Gerstenhaber, Silvano Raia, Peter I. Lelkes, Mayana Zatz

**Affiliations:** 1 Human Genome and Stem-Cell Research Center (HUG-CEL), Department of Genetics and Evolutionary Biology, Institute of Biosciences, University of São Paulo, São Paulo, SP Brazil; 2 Brazilian Biosciences National Laboratory (LNBio), Brazilian Center for Research in Energy and Materials (CNPEM), Campinas, SP 13083-970 Brazil; 3 Laboratory of Cellular Biology, Department of Pathology, Medical School, University of São Paulo, São Paulo, SP Brazil; 4 Department of Bioengineering, Temple University, Philadelphia, PA USA; 5 Department of Surgery, Medical School, University of São Paulo, São Paulo, SP Brazil

**Keywords:** Organoid, Liver, iPS, Hepatocyte, 3D culture

## Abstract

**Background:**

Liver organoid technology holds great promises to be used in large-scale population-based drug screening and in future regenerative medicine strategies. Recently, some studies reported robust protocols for generating isogenic liver organoids using liver parenchymal and non-parenchymal cells derived from induced pluripotent stem cells (iPS) or using isogenic adult primary non-parenchymal cells. However, the use of whole iPS-derived cells could represent great challenges for a translational perspective.

**Methods:**

Here, we evaluated the influence of isogenic versus heterogenic non-parenchymal cells, using iPS-derived or adult primary cell lines, in the liver organoid development. We tested four groups comprised of all different combinations of non-parenchymal cells for the liver functionality in vitro. Gene expression and protein secretion of important hepatic function markers were evaluated. Additionally, liver development-associated signaling pathways were tested. Finally, organoid label-free proteomic analysis and non-parenchymal cell secretome were performed in all groups at day 12.

**Results:**

We show that liver organoids generated using primary mesenchymal stromal cells and iPS-derived endothelial cells expressed and produced significantly more albumin and showed increased expression of CYP1A1, CYP1A2, and TDO2 while presented reduced TGF-β and Wnt signaling activity. Proteomics analysis revealed that major shifts in protein expression induced by this specific combination of non-parenchymal cells are related to integrin profile and TGF-β/Wnt signaling activity.

**Conclusion:**

Aiming the translation of this technology bench-to-bedside, this work highlights the role of important developmental pathways that are modulated by non-parenchymal cells enhancing the liver organoid maturation.

**Electronic supplementary material:**

The online version of this article (10.1186/s13287-019-1367-x) contains supplementary material, which is available to authorized users.

## Background

Liver organogenesis can be in part recapitulated in part by using organoid technology [1]. The combination of defined ratio of parenchymal progenitor cells (i.e., hepatoblast) and non-parenchymal cells (NPC) (i.e., endothelial cells and mesenchymal cells) recreates a cellular microenvironment akin to the early stages of liver bud development and allows for spontaneous tissue formation [2]. The first attempts to bioengineer complex liver organoids (LOs) used hepatoblasts derived from human pluripotent stem cells (iPS) in conjunction with primary human NPC, such as human umbilical cord-derived endothelial cells (HUVEC) and adipose tissue-derived mesenchymal stem cells (MSCs), all derived from different donors [1].

NPC contribute to liver development and homeostasis by secreting growth factors (e.g., TNF-α, IL-6, HGF, TGF-β, and BMP2, 4 and 6) that regulate hepatocyte proliferation, DNA synthesis, and hepatic cord formation [3–5]. Asai and collaborators [6] showed the distinct contributions of primary lineages of endothelial cells (ECs) and MSC secretome in LO development in vitro*.* More recently, some other groups reported a series of combined protocols for generating isogenic LOs obtained from whole iPS-derived cells, obtained from the same donor, or by using primary NPCs from the same donor [7–9]. Takebe and collaborators [7] successfully generated LOs from human donors that could potentially be applied for high-throughput personalized screening of liver toxicity.

However, large-scale differentiation of iPS into multiple cell lineages is challenging in terms of cost and efficiency as opposed to primary cell lineages. As a caveat, the use of standard commercial non-parenchymal cell lines will yield human LOs that are chimeric in nature. Here, we propose to evaluate the effects of applying liver NPCs derived from iPS-derived fetal-like cells versus adult primary NPC cell lines to LO development and functionality.

## Methods

### iPS generation and culture and primary adult cell culture

Induced pluripotent stem cells (iPSs) were generated from three healthy human donors (F9048 = male, 26; F8799 = female, 28; F7405 = male, 23), as previously described [10]. The reprogramming and cell culture protocol were described in Additional file 1: methods. Differentiation protocols and human primary adult cell culture methods were described in Additional file 1: methods.

### Liver organoid

Prior to cell seeding, Matrigel was diluted 1:1 on ice with cold EGM-2 and dispensed at 380 μL/well in a 24-well plate. Gelling was achieved by incubation in 37^°^C for at least 30 min. A mixture of iPS-derived cells (1 × 10^6^ hepatoblast, 8 × 10^5^ ECs, and 2 × 10^5^ MSCs, as per Takebe et al. [1]) was centrifuged for 5 min at 300×*g* and resuspended in 2 mL of LO culture media (composed of 1:1 EGM-2/hepatocyte differentiation media, see Additional file 1: methods). The cell mixture was seeded on top of the Matrigel bed. Media was changed every other day. In order to assess the rate of mesenchymal condensation, pictures of the wells were taken every 12 h. The confluent cell layer and the total covered area progressive condensation over time was evaluated using ImageJ software.

### Proteomics

Proteomic sample processing and analysis followed a previously published protocol [11]. For detailed information, see Additional file 1: methods section. Pathway annotation of protein IDs was performed using the comprehensive EnrichR gene set enrichment analysis web server [12, 13], applying Reactome [14] and Panther [15] categorization with the significance threshold set at *p* < 0.05. Interactome analysis was performed using String [16] with k-means clustering in three groups.

### Statistical analysis

Statistical analyses to assess LO functional analysis and development quality (Figs. 2 and 4) were performed using one-way ANOVA with Tukey’s post-test. For all other statistical analyses, Student’s two-tailed *t* tests were used for pairwise comparisons. Data are presented as means ± SEM, or mean of at least three independent experiments, with at least two technical replicates. For the proteomics analyses, statistical tests were performed using Students *t* test, using Perseus software, and pathway enrichment analysis using EnrichR. Values of *p* < 0.05 were considered significant. GraphPad Prism software was used to perform all other statistical analyses.

## Results

### iPS cell differentiation

**Fig. 1:**
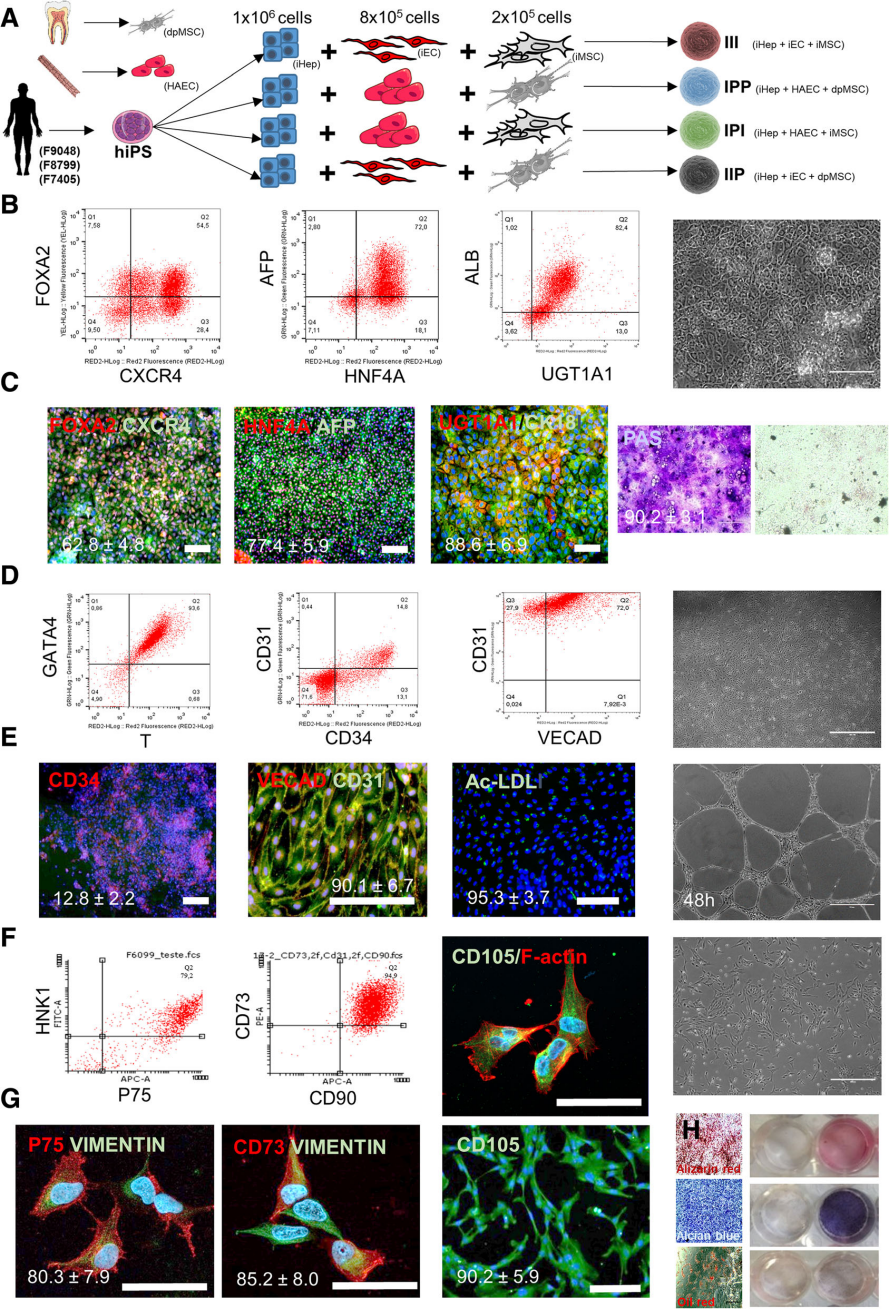
iPS cell differentiation. **a** Graphical summary of experimental groups and design. **b** Step-wise flow cytometry characterization of hepatocyte differentiation in vitro. Representative image of gated analyses for FOXA2^+^/CXCR4^+^ at day 3, AFP^+^/HNF4A^+^ at day 9, UGT1A1^+^/ALB^+^ at day 18, and phase-contrast image of cellular morphology at day 18. **c** IF staining for FOXA2/CXCR4 at day 3, AFP/HNF4A at day 9, and UGT1A1/ALB and PAS staining of hepatocytes at day 18 and confluent fibroblast culture PAS staining negative control (*n* = 3, biological replicates; data displayed as mean ± SEM, bar = 50 μm). **d** Step-wise flow cytometry characterization of endothelial differentiation in vitro. Representative images of gated analyses for BRACHYURY^+^/GATA4^+^ at day 2, CD34^+^/CD31^+^ at day 6, VECAD^+^/CD31^+^ at day 10, and phase-contrast image of cellular morphology at day 10. **e** IF staining for CD34 at day 2, VECAD/CD31 and Ac-LDL uptake at day 10, and angiogenesis assay at day 12 (*n* = 3, biological replicates; data displayed as mean ± SEM, bar = 50 μm). **f** Step-wise flow cytometry characterization of iNCC-MSC differentiation in vitro. Representative image of gated analyses for P75+/HNK1+ at day 18 and CD73+/CD90+ and IF staining for CD105/F-Actin and phase-contrast image at day 28. **g** IF staining for P75/VIMENTIN at day 18, CD73/VIMENTIN and CD105 at day 28, and representative images for MSC differentiation assay after 28 days of induction and stained for Alizarin Red, Alcian Blue, and Oil red (*n* = 3, biological replicates; data displayed as mean ± SEM, bar = 50 μm)

Aiming a broad applicability of our studies and reproducibility of the results, we carried out the experiments with three independent iPS cell lines. All data shown in Fig. 1 are related to cell line F9048, and similar results were observed with the other cell lines (Additional file 1: Figure S1C). Figure 1a summarizes the groups tested in this study, using different combinations of NPCs with iPS-derived hepatocytes. Here, we have used “I” to indicate cells derived from iPS and “P” to indicate primary cell lines. Isogenic LO (i.e., containing all three cell lines derived from the same iPSs) is referred to as III. When using NPC derived from primary human cell lines, the group is referred to as IPP. When using human aortic endothelial cells (HAECs) and iPS-derived MSC, the group is referred as IPI. Finally, when using iPS-derived EC and dental pulp-derived MSC (dpMSC), the group is referred as IIP.

The hepatic differentiation potential was evaluated and characterized in vitro. Figure 1b shows representative images of flow cytometric analyses. After 3 days of differentiation, 58.5 ± 4.7% (*n* = 3) of cells were CXCR4^+^/FOXA2^+^ (definitive endoderm). Despite starting out with a heterogeneous population, at day 9 of differentiation, a majority of cells (78 ± 5.8%, *n* = 3) expressed hepatic progenitor markers, such as HNF4A and AFP. After terminal hepatocyte differentiation, 74.3 ± 7.1% (*n* = 3) of the cells expressed the hepatic markers ALB and UGT1A1 (Fig. 1b). Phase-contrast image showed homogenous hepatocyte morphology in a monolayer culture. Representative images of immunofluorescence (IF) staining for each step of hepatic differentiation are shown in Fig. 1c. After 3 days, 62.8 ± 4.8% (*n* = 3) of cells were double positive for FOXA2 and CXCR4. After 9 days, 77.4 ± 5.9% (*n* = 3) were positive for HNF4A and AFP, and at day 18, 88.6 ± 6.9% (*n* = 3) of cell population were positive for ALB and UGT1A1. Also, at day 18, 90.2 ± 3.1% (*n* = 3) cells stained positive for Periodic acid-Schiff (PAS) (Fig. 1c).

For assessing endothelial differentiation, the iPS-derived cells were evaluated stepwise throughout the differentiation protocol. Figure 1d shows representative images of the flow cytometric analyses. The first step of the differentiation protocol (day 2) resulted in homogenous mesodermal differentiation, as inferred from the nearly ubiquitous co-expression of Brachyury T and GATA4 (92.4 ± 3.7, *n* = 3). However, at the end of endothelial differentiation (day 7), only 13.1 ± 2.7% of the cell population was double positive for endothelial markers CD34 and CD31. After magnetic sorting of the CD31+ cells and seeding a 60% confluent cell culture, the great majority of cells were positive for CD31 and a varying percentage of them were also positive for VECAD (48.6 ± 22.8%, *n* = 3). At day 7 of endothelial differentiation, 12.8 ± 2.2% of cells were positive for CD34. After cell sorting, 90.1% ± 6.7% of cells were double positive for CD31 and VECAD (Fig. 1e). IF staining was performed in 90% confluent culture, which could explain the difference observed in flow cytometry analysis. ECs took up acetylated LDL (95.3 ± 3.7%, *n* = 3) and were able to generate capillary-like tubular structures in the Matrigel angiogenesis assay (Fig. 1d, e).

For mesenchymal differentiation, flow cytometric analysis indicated that the majority of iNCC cells expressed HNK1 and P75 (79.0 ± 3.1%, *n* = 3). Following mesenchymal terminal differentiation, essentially all the cells showed a typical MSC morphology, were positive for the majority of cell population which expressed CD73 and CD90 (95.1 ± 1.8%, *n* = 3) and CD105 (Fig. 1f). Also, iNCC staining revealed that the majority (80.3 ± 7.9%) of the cells were double positive for P75 and Vimentin. Similarly, most (85.2 ± 8.0%) iNCC-derived MSCs were double positive for CD73 and Vimentin (Fig. 1g). Additionally, 90.2 ± 5.9% of these MSCs were positive for CD105 (Fig. 1g). Finally, we tested the ability of the iNCC-derived MSC to differentiate into osteogenic, chondrogenic, and adipogenic lineages. Figure 1h shows representative, low-magnification bright-field images and photographs of cell culture wells, and negative controls, stained for Alizarin Red, Alcian Blue, and Oil Red, respectively, after 28-day exposure to the various MSC differentiation induction protocols.

### Liver organoid functional analysis

**Fig. 2:**
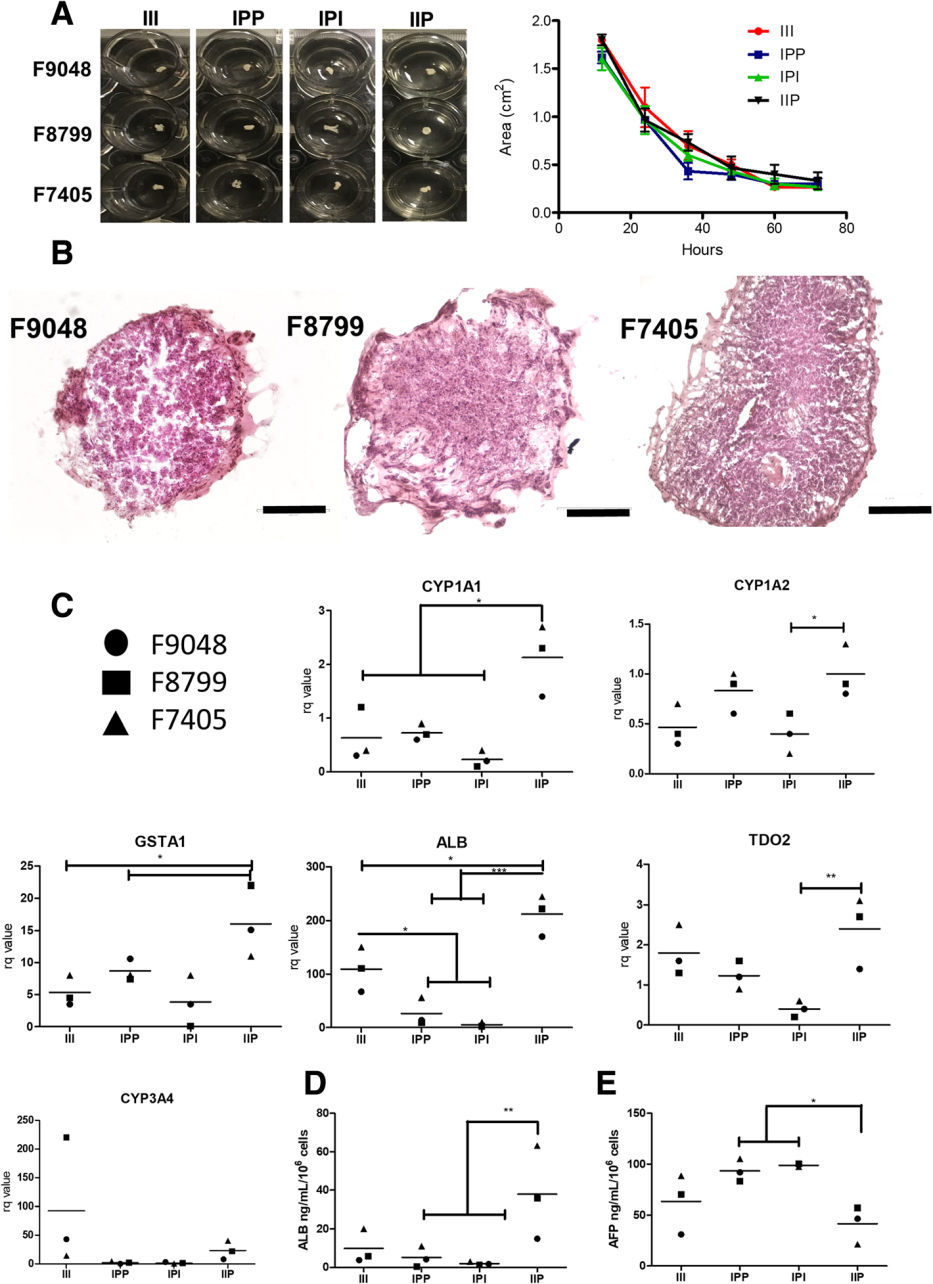
Liver organoid functional analysis. **a** Representative image of liver organoid culture of all cell lines and tested conditions after 72 h of mesenchymal condensation in a 24-well plate and area analysis overtime (*n* = 3, biological replicates; data displayed as mean ± SEM). **b** HE staining for III groups of all cell lines (bar = 50 μm). **c** RT-qPCR gene expression analysis of hepatic markers at day 12, normalized with 2D cultured iPS-derived hepatocyte at day 18 of differentiation protocol. **d** Normalized albumin secretion at day 6 and day 12. **d** Urea secretion at days 6 and 12. **e** AFP secretion at day 12 (*n* = 3, biological replicates; data displayed as great mean and individual scatter plots; one-way ANOVA with Tukey’s post hoc, *p < 0.05, **p < 0.01, ***p < 0.01)

Liver organoid (LO) formation was evaluated by assessing the tissue condensation rate (i.e., mesenchymal condensation rate) [7]. As seen in Fig. 2a, the rate of mesenchymal condensation was essentially identical for all cell lines and all experimental groups. No apparent morphological differences were observed in histological staining in the three cell lines and between groups (Fig. 2b shows representative H&E images of the III group). No difference in cellular distribution was observed in IF tissue staining for hepatic, endothelial, and MSC markers (Additional file 1: Figure S1I). LO express important MRP1, an important basal membrane transporter, and were able to perform basolateral transportation, as shown by CDFDA staining (Additional file 1: Figure S1I). Analysis of gene expression by RT-qPCR after 12 days of LO culture revealed increased expression of important hepatic phase I xenobiotic biotransformation enzymes in IIP, such as *CYP1A1* and *CYP1A2*, but not *CYP3A4*, and also an increased expression of the phase II enzyme *GSTA1*. Although CYP3A4 gene expression was not altered, enzymatic activity was significantly higher in group IIP (Additional file 1: Figure S1G). The data also indicate an increased expression of important hepatic maturation markers, such as *ALB* and *TDO2*. ELISA analysis of the LO culture supernatants revealed a significantly increased (6.7-fold) amount of secreted albumin by day 12 in the IIP group, as compared to other groups (Fig. 2d). Also, for the same time point, we observed a significant reduction of AFP secretion in the supernatants of group IIP, as compared to groups IPP and IPI (Fig. 2e). No statistical differences were observed at day 6 (Additional file 1: Figure S1E). We did not observe any differences in A1AT and LDH media production in all groups and in different time points (Additional file 1: Figure S1E).

### LO developmental pathway analysis

**Fig. 3:**
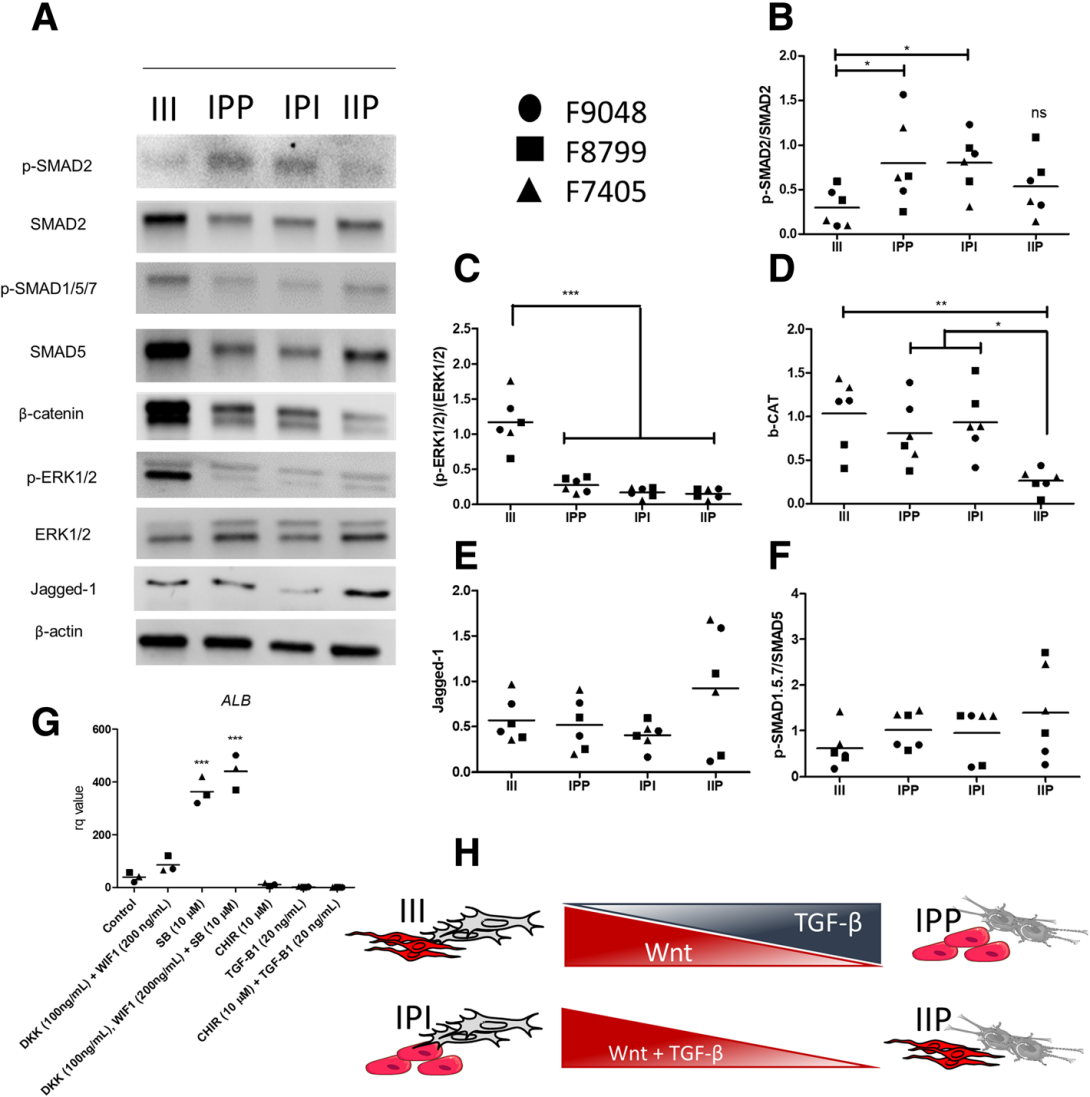
Liver organoid cell signaling analysis. **a** Representative western blotting gels for all evaluated proteins (**b**) pSMAD2/SMAD2 analysis. **c** p-ERK1/2/ERK1/2 analysis. **d** β-Catenin analysis. **e** Jagged-1 analysis. **f** p-SMAD1.5.7/SMAD1.5.7 analysis. **g** Albumin gene expression after IPP organoid culture in presence of TGF-β and Wnt antagonist and agonist for 10 days. Data was normalized with untreated iPP organoid. **h** Graphical representation of western blotting results (*n* = 6, biological replicates; data displayed as great mean and individual scatter plots; one-way ANOVA with Tukey’s post hoc, *p < 0.05, **p < 0.01, and ***p < 0.001)

Western blotting analysis of important cell signaling pathways related to liver development is displayed in Fig. 3a (experiment performed twice, independently). Densitometry analysis revealed significantly reduced activity (i.e., reduced ratio of phosphorylated/total protein) of SMAD2 (Fig. 3b) in III, as compared to IPP and IPI, but not to IIP. ERK1/2 activity was increased exclusively in III (Fig. 3c). β-Catenin expression was reduced in IIP, as compared to all groups (Fig. 3d). No statistically significant differences across all groups were found in the activities of Jagged-1 and SMAD1.5.7 (Fig. 3e, f). To confirm the western blotting findings, we performed an additional set of experiments using combinations of Wnt and TGF-β agonists (i.e., CHIR99021 and TGF-β1, respectively) and antagonist (i.e., DKK, WIF1, and SB431542, respectively) during LO maturation (Fig. 3g) for 10 days. Combinations of both agonists, 10 μM CHIR99021 + 20 ng/mL of TGF-B1, significantly reduced the albumin gene expression, while the antagonists, 100 ng/mL of DKK, 200 ng/mL WIF1 plus 10 μM SB431542, significantly increased albumin gene expression (Fig. 3g).

### Proteomics

**Fig. 4:**
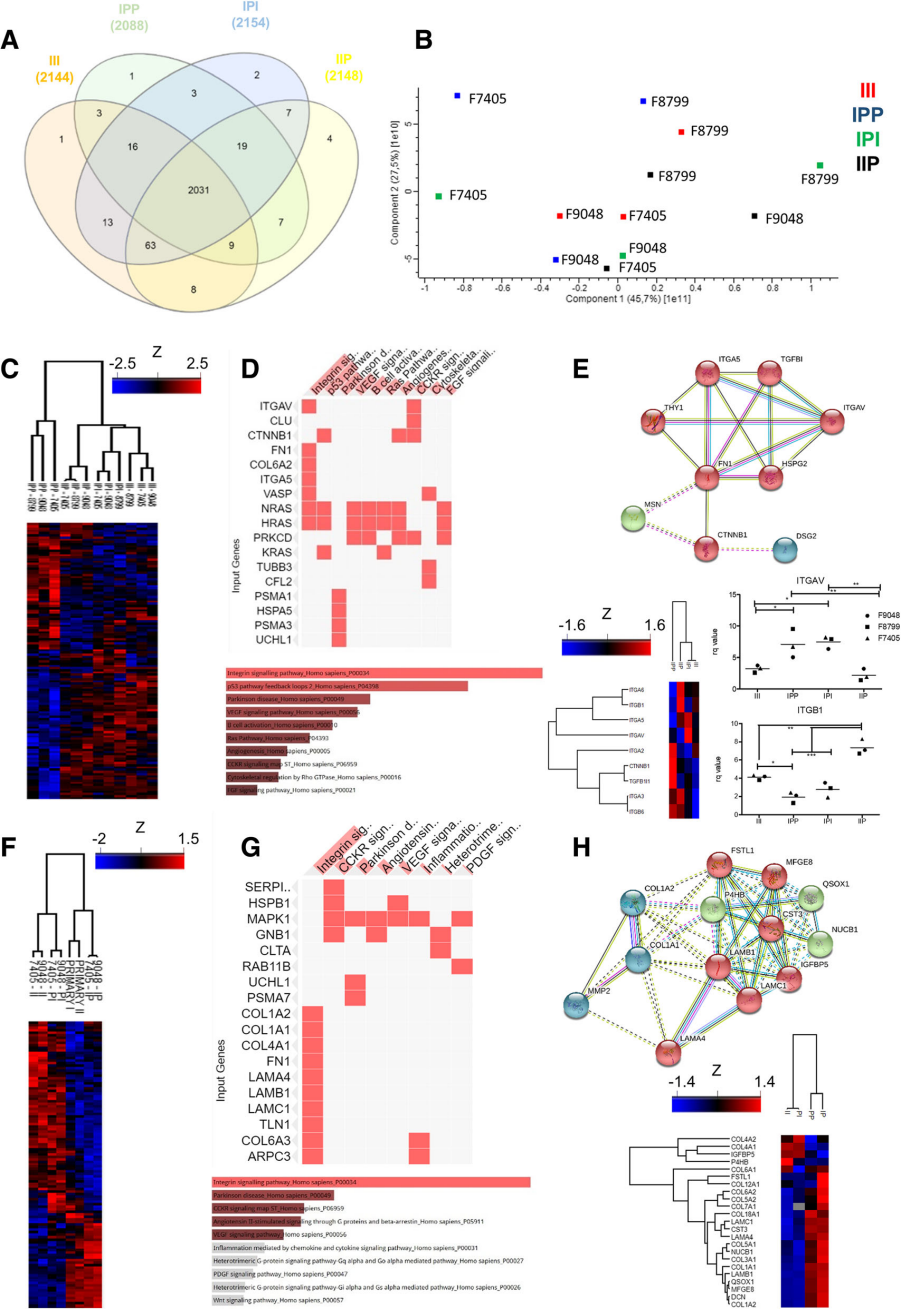
Liver organoid proteomic analysis. **a** Venn diagram of all identified proteins in all groups. **b** Principal component analysis of all tested samples. **c** Heat map of *Z*-scores from differentially expressed protein IDs in LO after one-way ANOVA test (*p* < 0.05). **d** Pathway enrichment analysis using EnrichR related to **c**. **e** String interactome graph of protein from the list from **c**, filtered by most enriched GO biological process, colored differently accordingly to k-means clustering. RT-qPCR for *ITGAV* and *ITGB1* (*n* = 3, biological replicates, normalized with 2D cultured iPS-derived hepatocyte at day 18 of differentiation protocol; data displayed as great mean and individual scatter plots; one-way ANOVA with Turkey’s post hoc, *p < 0.05 and **p < 0.01). Filtered heat map for TGF, Wnt, and integrin signaling. **f** Heat map of *Z*-scores from differentially expressed protein IDs in LO secretome after one-way ANOVA test (*p* < 0.05). **g** Pathway enrichment analysis using EnrichR related to **f**. **h** String interactome graph of the protein list from **f**, filtered by most enriched GO biological process and filtered heat map for proteins related to ECM organization and IGF are colored differently accordingly to k-means clustering

Proteomic profiling revealed significant differences in LO groups at day 12. Of the approximately 2100 proteins identified in each group, the vast majority of the protein IDs (2031) were identified (Fig. 4a). Principal component analysis showed the absence of clustering among all samples tested (Fig. 4b). Figure 4c shows a heat map of differentially expressed proteins, when applying ANOVA with *p* < 0.05 threshold. Hierarchical clustering shows that the most different group was IPP, while the most similar among all groups were III and IPI. Figure 4d highlights the integrin signaling as the most enriched pathway identified. Figure 4e shows the interactome from ANOVA-tested, differentially expressed proteins, filtered by the most enriched GO according to biological function. The two major clusters of nodes are located around FN1 and the integrin alpha subunits V and 5, first shell of interactors, and TGFBI (i.e., TGF-β induced protein) and CTNNB1 (i.e., β-catenin), corroborating western blotting findings. Gene expression analysis of *ITGAV* by RT-qPCR confirmed reduced expression in groups III and IIP, as opposed of what was observed in *ITGB1*. Secretome analysis of 2D co-culture of NPCs is displayed in Fig. 4f–h. Figure 4f shows the heat map generated by ANOVA with *p* < 0.05 thresholds. Hierarchical clustering shows that IP is more similar to primary NPCs and that II is more similar to PI. Pathway enrichment analysis of the secretome of NPCs (Fig. 4g) highlights the role of integrins/extracellular matrix (ECM), TGF-β, and IGF, which are filtered and displayed in the heat map in Fig. 4h. The interactome of ANOVA-tested, differentially expressed proteins, filtered for ECM and IGF signaling, is shown in Fig. 4h.

## Discussion

A major aim of the present study was to elucidate the role and efficacy of NPCs, derived either from iPS or from primary cell culture, in the development and functionality of LOs. Previous reports showed that it is possible to generate isogenic LOs using either primary or iPS-derived liver NPCs [8, 9]. However, the impact of using such distinct NPC sources in LO maturation has never been addressed.

Here, we show that all human iPS cell lines used in the present study were characterized by flow cytometry and gene expression (Additional file 1: Figure S1A and B). We successfully differentiated all three iPS cell lines towards hepatoblasts, arterial ECs, and iNCC-derived MSCs (Fig. 1 and Additional file 1: Figure S1C). The differential contribution, if any, of arterial, venous, or lymphatic ECs in the development of LO remains to be elucidated. Thus, we used iPS-derived arterial ECs, with commercially available HAECs as the correspondent adult cell line. For mesenchymal cells, we used iNCC-derived MSC, with dpMSC as the primary adult cell counterpart. Although there are many available protocols to differentiate iPS towards MSC, the vast majority of them lack proper comparison to the specific adult tissue MSC and are reported as a general embryonic or mesodermal MSC differentiation [17–19]. Since MSCs have tissue-specificity functionality, aiming to properly compare the use of primary versus iPS-derived MSC in LO maturation, we needed to compare matching tissue-derived primary MSC to iPS-differentiated tissue analog. Additionally, it is well known that iPS-derived MSCs obtained from different intermediated germ lines have different properties [18]. Our group recently reported a protocol to generate cranial facial MSC (obtained from iPS-derived neural crest cells) that closely resembles MSC located at the dental pulp [10]. Here, we were able to compare matching tissue specificity of MSCs (i.e., primary vs iPS-derived) in the LO maturation process.

We succeeded in deriving functional hepatoblasts, as assessed by the expression of HNF4A and AFP. The potential of our hepatoblasts to differentiate into hepatocyte was inferred from the expression pattern of mature hepatic markers such as UGT1A1 and CK18 and by PAS staining (Fig. 1b, c).

Arterial endothelial cell commitment was inferred from the expression of general endothelial markers, such as *CD31* and *VECAD*, together with that of specific arterial EC markers such as *NOTCH4* and the very low levels of expression of *PDPN* and *EPHB4*, which are markers of lymphatic and venous phenotype, respectively (Additional file 1: Figure S1B). The functionality of our iPS-derived ECs was demonstrated by their ability to take up acetylated LDL and to generate capillaries in Matrigel (Fig. 1d, e).

Neural crest differentiation was confirmed by the expression of HNK1 and CD75 (Fig. 1f). iNCC-derived MSC expressed stromal-mesenchymal markers such as CD105, CD90, and CD73 (Fig. 1f, g). Following in vitro induction, the mesenchymal differentiation was confirmed by the osteogenic staining with Alizarin Red (calcium deposits), chondrogenic staining with Alcian Blue (glycosaminoglycans), and adipogenic staining with Oil Red (lipid droplets) (Fig. 1h). This result indicates that we have successfully derived competent multipotent mesenchymal cells, and not fibroblasts.

LO generation was compared between all tested groups. No differences were observed in the mesenchymal condensation rate and in the morphology between all tested groups (Fig. 2a, b). Even though we noted some intrinsic variations between the tested cell lines, our RT-qPCR data at day 12 (Fig. 2c) revealed that important genes related to hepatic xenobiotic metabolism of phase I (i.e., *CYP1A2* and *CYP1A1*) and II (i.e., *GSTA1*) were overexpressed in IIP. Even though CYP3A4 RT-qPCR data showed no significant differences, enzymatic activity levels were significantly higher in group IIP. These data suggest that the hepatic metabolic rate was higher in the presence of dpMSC associated with iPS-derived ECs (i.e., group IIP) and reduced in the presence of adult arterial ECs. ALB and TDO2 gene expression were also significantly elevated in group IIP, which suggests increased hepatic maturation. In addition, IIP produced more albumin at day 12 (Fig. 2c), with reduced AFP gene expression and secretion, as compared to IPI and IPP, but not to III. A concomitant increase in albumin and reduction of AFP secretion is one of the most important hallmarks of hepatocyte maturation [20–22]. The secretion of AA1T and LDH was not altered between the groups and at the time points tested (Additional file 1: Figure F).

To evaluate the influence of NPC in key signaling pathways relevant to LO formation, we performed a series of western blots (Fig. 4a) [23–29]. The protein activity analysis revealed that the III and IIP groups exhibited significantly lower activity of TGF-β (Fig. 4b). Also, the IIP group showed significantly reduced Wnt activity (Fig. 4d), while the III group exhibited increased ERK1/2 activity (Fig. 4c). Activated ERK1/2 inhibits GSK3B through c-Met or IGF receptor signaling [29], which could explain high β-catenin in III. No differences were observed in the signaling of Notch and BMP4 (Fig. 4e, f). Figure 4h compiles the information obtained from our western blot analyses. TGF-β inhibition increased LO albumin production in vitro by inducing hepatoblast differentiation towards hepatocytes, thereby suppressing cholangiocyte differentiation [30]. Also, TGF-β is positively correlated with lower O_2(g)_ levels and activation of HIF1A during liver organogenesis [31]. In addition, Wnt signaling inhibition is known to control hepatocyte differentiation in 3D culture [32]. The combined inhibition of Wnt and TGF-β significantly increases the expression of albumin (more pronounced by TGF-β inhibition), as opposed of what was observed, when these two pathways were activated (Fig. 3g).

In order to confirm and evaluate the impact of previous western blotting analysis, we performed a proteomic profiling of the various LOs and of the NPC culture secretome. We observed intrinsic and differential protein expression patterns assigned by differential contribution of NPC to LO development (Fig. 4a–e). While sharing most of protein IDs (Additional file 1: Figure S1A), the tested NPC composition significantly influenced LO developmental pathways (Additional file 1: Figure S1B).

Most differentially enriched pathways were related to integrin signaling (Fig. 4c–e). The fibronectin receptor ITGAV (integrin receptor αV) was one of the major hits identified in our pathway enrichment analysis, as well as the integrin alpha subunit 5 (ITGA5). ITGAV expression is induced by TGF-β and acts promoting epithelial-mesenchymal transition [33] and fibrosis [34]. Integrin subunits α5 and β1 are necessary for bile duct epithelial tract formation during liver development [35]. Also, integrin β1 is important for sustaining hepatocyte viability in native ECM and has been implicated in liver regeneration [36, 37]. Importantly, specific integrin subunit combinations during liver organogenesis, such as α5β1, help to generate the different hepatic structures and are influenced by surrounding sinusoids, vascular development, and local ECM [38]. In our secretome analyses of the NPC cultures, Decorin, a well-known endothelial-produced repressor of liver fibrosis and local inhibitor of TGF-β [39] and c-Met [40], was significantly increased in group IIP (Fig. 4h). The reduced secretion of IGFBP5 by dpMSC (Fig. 4h), a MAPK signaling activator overexpressed during fibrosis [41], could explain the high ERK1/2 in III and reduced β-catenin in IIP. dpMSC produces more ECM, except for collagen type IV, but their role in LO maturation remains unclear.

Collectively, the expression of integrin β1, but not αV, and reduced TGF-β and Wnt signaling observed in the combination of iPS-derived EC and dpMSC, might explain the observed differences in hepatocyte function in various LOs. Our data suggests that high TGF-β activity induced by HAEC (Fig. 3b) increased expression of ITGAV and induced ECM remodeling that impairs hepatocyte maturation. Additionally, we suggest that Wnt signaling repression in IIP is due to reduced secretion of IGFBP5 by dpMSC.

## Conclusion

Our data indicates that reduced activity of TGF-β and Wnt contributes for the increased albumin secretion and hepatic function observed in the combination of dpMSC and iPS-derived ECs as NPCs. These differential growth factor stimuli generate substantial changes in integrin and ECM profiles that regulate liver development. In translational terms, this work provides important insights for assessing future strategies to advance organoid technologies aiming at high-throughput drug screening platforms and regenerative therapy approaches.

## Additional file

Additional file 1: Supplementary tables, methods, and figures (DOCX 1729 kb)

## Data Availability

All data provided in this study are available within the article and its additional information files or accordingly to section “Data availability”.

## Abbreviations

A1AT: Alpha 1 antitrypsin
AFP: Alpha fetoprotein
ALB: Albumin
BMP: Bone morphogenetic protein
CXCR4: C-X-X motif receptor 4
CYP: Cytochrome P450
DKK: Dickkopf-related protein
dpMSC: Dental pulp-derived MSC
ECs: Endothelial cells
EGM: Endothelial growth media
ELISA: Enzyme-linked immunosorbent assay
FN1: Fibronectin
FOXA2: Forkhead box A2
GATA4: GATA binding protein 4
GSTA1: Glutathione S-transferase alpha 1
HAECs: Human aortic endothelial cells
HGF: Hepatocyte growth factor
HIF1A: Hypoxia induced-factor 1A
HNF4A: Hepatocyte nuclear factor 4 alpha
HNK1: Human natural killer-1
HUVEC: Human umbilical vein endothelial cells
IF: Immunofluorescence
IGF: Insulin-like growth factor
III: Organoid comprised by iPS-derived NPC
IIP: Organoid comprised by iPS-derived endothelial cells and dpMSC
IL-6: Interleukin 6
iNCC: iPS-derived neural crest cells
IPI: Organoid comprised by iPS-derived MSC and HAEC
IPP: Organoid comprised by adult primary NPC
iPS: Induced pluripotent stem cells
LDH: Lactate dehydrogenase
LDL: Low-density lipoprotein
MSC: Mesenchymal stem cells
NPC: Non-parenchymal cells
P75: Nerve growth factor receptor
PAS: Periodic acid of Schiff
PDPN: Podoplanin
RT-qPCR: Reverse transcriptase quantitative polymerase chain reaction
SEM: Standard error of mean
TDO2: Tryptophan 2,3-dioxygenase
TGF-β: Transforming growth factor beta
TNF-α: Tumor necrosis factor alpha
UGT1A1: UDP glucuronosyltransferase family 1 member A1
VECAD: Ve-cadherin
WIF1: Wnt inhibitory factor 1
