# Supplementary material for: Adult and iPS-derived non-parenchymal cells regulate liver organoid development through differential modulation of Wnt and TGF-β
Source: Stem Cell Res Ther. 2019 Aug 15;10:258. doi: 10.1186/s13287-019-1367-x (PMC6694663; doi:10.1186/s13287-019-1367-x)

**Supplemental information**

1. **Supplementary Tables**

**Supplementary Table 1 – Antibodies used for IF staining**

| **Antibody** | **Catalog number** | **Host Specie** | **Assay dilution** |
| --- | --- | --- | --- |
| anti-AFP | ab133617 | Rabbit | 1/100 |
| Anti-CD105 | ab156756 | Mouse | 1/100 |
| Anti-CD31 | ab119339 | Mouse | 1/100 |
| Anti-CD34 | ab81289 | Rabbit | 1/100 |
| Anti-CD45 | ab10558 | Rabbit | 1/100 |
| Anti-CD73 | ab175396 | Rabbit | 1/100 |
| Anti-CK18 | ab32118 | Rabbit | 1/100 |
| anti-CXCR4 | ab124824 | Rabbit | 1/100 |
| Anti-FOXA2 | ab60721 | Mouse | 1/100 |
| Anti-HNF4A | ab41898 | Mouse | 1/100 |
| Anti-MRP1 | ab24105 | Mouse | 1/100 |
| Anti-OCT3/4 | ab19857 | Rabbit | 1/100 |
| Anti-P75 | ab52987 | Rabbit | 1/100 |
| anti-SSEA4 | ab16287 | Mouse | 1/100 |
| Anti-UGT1A1 | ab129729 | Mouse | 1/100 |
| Anti-VECAD | ab33168 | Rabbit | 1/100 |
| Anti-Vimentin | ab8978 | Mouse | 1/100 |
| Donkey anti-Mouse Alexa 546 | A10036 | N/A | 1/1000 |
| Goat anti-Mouse Alexa 488 | 11001 | N/A | 1/1000 |
| Goat anti-Rabbit Alexa 488 | 11034 | N/A | 1/1000 |
| Goat anti-Rabbit Alexa 546 | A11010 | N/A | 1/1000 |

**Supplementary Table 2 - Antibodies used for flow-cytometry staining**

| **Antibody** | **Catalog number** | **Host Specie** | **Assay dilution** |
| --- | --- | --- | --- |
| anti-AFP | PA5 21-004 | Rabbit | 1/100 |
| Anti-UGT1A1 | ab194697 | Rabbit | 1/100 |
| Anti-HNF4A | ab41898 | Mouse | 1/100 |
| Anti-ALB | ab106582 | Chicken | 1/100 |
| Anti-CD105 | ab156756 | Mouse | 1/100 |
| Anti-CD34 | ab81289 | Rabbit | 1/100 |
| Anti-CD31 | ab119339 | Mouse | 1/100 |
| Anti-VECAD | ab33168 | Rabbit | 1/100 |
| Anti-Brachyury T | ab140661 | Mouse | 1/100 |
| Anti-GATA2 | LS-C166381 | Rabbit | 1/100 |
| Anti-HNK1 | ab187274 | Mouse | 1/100 |
| anti-Mouse Alexa 647 | ab 150115 | Goat | 1/1000 |
| anti-Rabbit Alexa 647 | ab150075 | Goat | 1/1000 |
| Goat anti-Mouse Alexa 488 | 11001 | Goat | 1/1000 |
| Goat anti-Rabbit Alexa 488 | 11034 | Goat | 1/1000 |
| Goat anti-Chicken Alexa 488 | A0001 | Goat | 1/1000 |
| Anti-P75 | ab52987 | Rabbit | 1/100 |

**Supplementary Table 3 - Antibodies used for Western Blotting**

| **Antibody** | **Catalog number** | **Host Specie** | **Assay dilution** |
| --- | --- | --- | --- |
| Anti Jaaged 1 | sc-390177 | Mouse | 1/1000 |
| Anti -p-SMAD2 | 138D4 | Rabbit | 1/1000 |
| Anti SMAD2 | #5339 | Rabbit | 1/1000 |
| Anti p-ERK1/2 | #4377 | Rabbit | 1/1000 |
| Anti ERK1/2 | #4695 | Rabbit | 1/1000 |
| Anti p-SMAD1.5.7 | #9511S | Rabbit | 1/1000 |
| Anti SMAD5 | #9517P | Rabbit | 1/1000 |
| Anti YAP/TAZ | #8418 | Rabbit | 1/1000 |
| Anti β-catenin | #9582P | Rabbit | 1/1000 |
| Anti β-actin | ab49900 | Mouse | 1/40000 |
| Anti-Rabbit HRP | 7074S | Goat | 1/5000 |
| Anti-Mouse HRP | 7076S | Goat | 1/5000 |

**Supplementary Table 4 – Primers sequences list**

| **Gene** | **Foward** | **Reverse** |
| --- | --- | --- |
| OCT3/4 | TCCCATGCATTCAAACTGAGG | CCA AAA ACC CTG GCA CAA ACT |
| EphB4 | ACGGGGTATCCTCCTTAGCC | GTACCTCTCGGTCAGTGGTG |
| Notch4 | GCACTGCCAGAGATCCTCAT | CCCTAGCTCTGCCTCACACT |
| Podoplanin | ACCAGTCACTCCACGGAGAAA | GGTCACTGTTGACAAACCATCT |
| NANOG | TGGACACTGGCTGAATCCTTC | CGTTGATTAGGCTCCAACCAT |
| CXCR4 | CAAGGCCCTCAAGACCACAG | TGTAGTAAGGCAGCCAACAGG |
| T | CTGGGTACTCCCAATGGGG | GGTTGGAGAATTGTTCCGATGA |
| PAX2 | CCAAAGTTCAGCAGCCTTTCC | ATTGGAGGCGCTGGAAACAG |
| LIN28 | AGCGCAGATCAAAAGGAGACA | CCTCTCGAAAGTAGGTTGGCT |
| CYP1A1 | CCTTGGAACCTTCCCTGATCC | GATCTTGGAGGTGGCTGAGGT |
| CYP1A2 | CTGGACTTCTTCCCCATCCTTC | GTTCTTGTCAAAGTCCTGATAGTGC |
| GSTA1 | CTGCCCGTATGTCCACCTG | AGCTCCTCGACGTAGTAGAGA |
| ALB | AATGTGCTGATGACAGGGCG | CGGCAATGCAGTGGGATTTT |
| TDO2 | AGAGCTGGCCTACCTGAAGA | GCCCCTCAGCGTACTGATTT |
| CYP3A4 | ATGGAAAAGTGTGGGGCTT | TCATGTCAGGATCTGTGATAGC |
| ITGAV | GCTGTCGGAGATTTCAATGGT | TCTGCTCGCCAGTAAAATTGT |
| ITGB1 | CCTGAGAGTGATGCTACTCCA | CACCCTGGTTGTGCCAAAAAT |

1. **Supplementary Methods**
   1. **iPS cell line generation and culture**

Cell reprogramming was performed according to [33], using an episomal vector system (pCXLE-hOCT3/4-shP53-F, addgene plasmid 27077; pCXLE-hSK, addgene plasmid 27078; pCXLE-hUL, addgene plasmid 27080) and using the Amaxa human CD34^+^ cells Nucleofection kit (Lonza), following the manufacturer’s recommendations.

CD71^+^-cells, which were isolated from donors’ peripheral blood mononuclear cells or dermal fibroblast were nucleofected. Three days after nucleoporation using Lonza Nucleofector 2b, cells were seeded on irradiated murine embryonic fibroblasts (Millipore – A24903) and cultured in maintenance medium, which was composed of DMEM/F12 supplemented with 2mM GlutaMAX-I, 0.1mM non-essential amino acids, 100 µM 2-mercaptoethanol, 20% of knockout serum replacement (all provided by Life Technologies), 10 ng/mL of bFGF (Peprotech), 0.25 mM NaB, 0.5 mM of valproic acid, 2 µM thiazovivin, 0.5uM PD 0325901 and 2µM SB 431542; all provided by Tocris Bioscience). Typical circular hiPS colonies were transferred to 35 mm circular dishes (Corning) coated with 10 µg/cm^2^ hESC-qualified Matrigel (Corning), incubated for 30 min at 37 C and cultured in Essential 8 medium (Gibco) supplemented with 100 μg/mL normocin (InvivoGen) with daily complete media change. All derived cell lines were checked periodically for mycoplasma contamination. In all procedures involving single-cell passaging were performed using Accutase for 3-5 min (Gibco), cells were seeded 2.5x10^4^ cells/cm^2^. After seeding, media were supplemented with 5 µM of Y-27632 (Sigma-Aldrich) upon seeding and maintained for 24h. Total DNA was extracted from iPS cultures using a NucleoSpin Tissue kit (Macherey-Nagel), following the supplier’s instructions. Multiplex ligation-dependent probe amplification (MLPA) analysis was performed with subtelomeric kits (P036 and P070; MRC-Holland) to detect chromosomal imbalances, as previously described [34].

**2.2 iPS cell differentiation**

Hepatocyte differentiation was performed accordingly previously published protocols) [35]. Briefly, single cell iPSs were seeded at cell density of 2.5x10^4^ cells/cm^2^ in Matrigel coated plates and culture for three days, with daily media changes, in endodermal induction media (RPMI 1640 (from Gibco), supplemented with 2% B27 (Gibco), 100 ng/mL of Activin A - R&D, 25 ng/mL of Wnt3a - R&D, 1% GlutaMAX – Gibco and 100 ug/mL of Normocin (Invivogen). Subsequently, the cells were cultured for 6 days in hepatoblast induction media, with media changes every other day (KO-DMEM (Gibco), supplemented with 20% knockout serum replacement (Life Technologies), 1% DMSO, 1% Glutamax and 100 ng/mL of normocin). Hepatocyte induction was carried for another 9 days, in media comprised of Hepatozyme (Gibco), supplemented with 10 ng/mL of HGF, 20 ng/mL (R&D) of Oncostatin M (R&D), 10 nM of hydrocortisone (Sigma-Aldrich) and 1% GlutaMAX, with media changes every other day.

Endothelial differentiation was carried out following the protocol of Sriram et al., (2015) [36], with few modifications. First, iPSs were plated at 1.5x10^4^ /cm^2^ and cultured for three days. For the next 5 days, basal media was comprised of Stempro-34 (Gibco) as basal media, supplemented daily with the following differentiation factors: On the first day, with 5 µM CHIR99021 and on the second day, basal media was supplemented with 50 ng/mL FGF-2. For the last three days, the basal media was supplemented with 50 ng/mL VEGF and 25 ng/mL BMP4. All recombinant agents were obtained from R&D systems. On the last day of differentiation, cells were detached using Accutase for 5 min and sorted magnetically using anti-CD31 microbeads (Miltenyi), following the manufacturer’s instructions. CD31^+^ cells were plated in 60mm Matrigel-coated dishes (Corning) and cultured for no more than 6 passages in serum-free human endothelial media (Gibco) supplemented with 10 ng/mL VEGF, 20 ng/mL FGF-2, 10 ng/mL EGF and 1% of human platelet lysate (Sigma).

Procedures for induced neural crest cell (iNCC) derivation were based on previously published methodology [10]. iPSs were seeded as single cells at 1× 10^4^ cells/cm^2^ onto 60-mm Matrigel-coated dishes and cultured in Essential 8 (E8) media. Two days post-seeding, the E8 media was changed to iNCC differentiation medium, composed of Essential 6™ Medium (Life Technologies) supplemented with 8 ng/ml FGF-2, 20 µM SB431542 (TOCRIS), 1 µM CHIR99021 and 100 µg/ml Normocin. The differentiation medium was changed a daily basis. After ∼2–4 days, neural crest-like cells were seen detaching from the borders of individual colonies. The cultures were split before reaching confluence, using Accutase. Cells were re-seeded into new 60-mm Matrigel-coated dishes in fresh iNCC differentiation medium. Cells were passaged using Accutase when they reached 80% confluency at 1:3 ratio whenever necessary, for 15 days. Differentiated iNCCs were cultivated for up to eight passages in iNCC differentiation medium, which was completely changed daily. Neural mesenchymal cell (nMSC) populations were obtained through culturing iNCCs with mesenchymal stem cell medium, as previously described [10]. In brief, iNCCs were seeded at 2×10^4^cells/cm^2^ onto non-coated 60-mm tissue culture dishes in nMSC medium (DMEM/F12 supplemented with 10% FBS, 2 mM GlutaMAX, 0.1 mM non-essential amino acids and 100 µg/ml Normocin. Cells were differentiated for 6 days and passaged with TrypLE™ Express (Life Technologies) when reached 80% confluency at 1:3 ratio. nMSC cultures were expanded in nMSC medium for up to 6 passages, with medium changes every 3 days.

- 1. **Human primary cell culture**

Human aortic endothelial cells (HAEC) were purchased from Thermo Fisher (C0065C, passage three). HAEC were seeded at 1.5x10^4^ cells/cm^2^ and cultured in EGM-2 (Lonza) with complete media changes every other day. Cells were split 1:3 when reached 80% confluency using Trypsin 0.05% (Life Technologies) and cultured for no longer then 10 passages. Dental-pulp MSC (dpMSC), obtained from our cell bank reservoir, have been previously described and fully characterized [37]. dpMSCs were cultured in DMEM/F12 supplemented with 10% FBS and 1% non-essential amino acids (Gibco).

**2.4 iPS-derived cells characterization**

**2.4.1 Immunofluorescence staining**

Cell cultures were fixed with 4% PFA for 20 minutes followed by permeabilization with 0.01% Triton X-100 for 30 minutes and blockage using 5% BSA in PBS for 1 h. After that, the cells were incubated overnight with primary antibodies (see supplementary Table 1) at 4 °C and subsequently incubated with secondary antibodies (see supplementary Table 1) for 1 h at room temperature. The final step was DAPI (Sigma) staining for 5 min at room temperature.

- - 1. **Flow cytometry**

Cells were stained for flow cytometry analysis to evaluate culture uniformity. Fixation and permeabilization were performed using a Fix & Perm Kit (Invitrogen) according to the manufacturer’s instructions. Cells were stained with conjugated primary antibody (see supplementary Table 2) for 45 min in an ice bath, washed with 1× PBS, and then analyzed by Guava Flow cytometer (Merck). The experiment was performed collecting at least 10,000 events per group. After data acquisition, further analysis was performed using FlowJo software. Gate was set using isotype control.

- - 1. **RT-qPCR**

Total RNA was isolated from cell culture using the RNeasy Mini Kit (Qiagen), following the manufacturer’s recommendations. Briefly, 1 μg of total RNA was converted into cDNA using Superscript II (Life Technologies) and oligo-dT primers according to the manufacturer’s specifications. RT-qPCR reactions were performed with Power SYBR Green Master Mix (Life Technologies). Fluorescence was detected using the Applied Biosystem 7500 Real-Time PCR System, under standard temperature protocol. Primer pairs were either designed with PrimerBLAST (http://www.ncbi.nlm.nih.gov/tools/primer-blast/) or retrieved from PrimerBank (http://pga.mgh.harvard.edu/primerbank/). Primers are listed in supplementary Table 4. Quantitative analyses were performed using a relative quantification curve with positive control and GAPDH as endogenous control.

- - 1. **ELISA**

Culture supernatant was collected every other day and store at -80C until further analysis. The samples were thaw and centrifuged at 300g for 10 min to remove cellular debris. ELISA was performed according to manufacturer’s instructions.

- - 1. **Western Blotting**

The whole-organoid protein lysates were extracted, and western blotting analysis was performed. Protein lysates (5 μg) were electroblotted on polyvinylidene difluoride (PVDF) membranes (GE Healthcare) and probed with respective primary antibodies overnight at 4 °C (Supplementary table 3). The next day, membranes were incubated with related peroxidase (HRP)-conjugated secondary antibodies (Supplementary table 3. Proteins were visualized using chemiluminescence substrate. Finally, blots were scanned using ImageQuant and analyzed by ImageJ software. Normalized band intensities against corresponding β-actin were calculated for precise comparison.

- 1. **Urea and LDH**

Culture supernatant was collected every other day. LDH assay (Thermo) and Urea quantification assay (Sigma) was performed accordingly to manufacture instructions.

- 1. **PAS staining**

Hepatocytes were fixed with 4% para-formaldehyde for 20 min at room temperature. Periodic acid of Schiff staining was performed following manufacturer's instructions (Sigma).

- 1. **Angiogenesis assay**

Matrigel (Corning) was diluted with EGM-2 (Lonza) 1:1 on ice. For coating a 24 well plate, 380ul of diluted Matrigel were add to each well and incubated for 30 min at 37C. Endothelial cells were plated at 5x10^5^ cells/well and cultured for 48h with EGM-2. The formation of a branched capillary bed was evaluated under phase contrast microscope.

- 1. **Ac-LDL uptake**

Alexa 488-labeled acetylated-LDL (Ac-LDL) (Thermo) was add to a final concentration of 10 ug/mL to the cell media and incubated for 2h in 37C. Plates were washed twice with DPBS and cell media was refilled. Cellular uptake was evaluated in a fluorescence microscope (Nikon).

- 1. **nMSC differentiation assay**

For induction of mesenchymal differentiation, 2,5x10^5^ nMSC per well were plated on 12 well plate (Corning). After 24h, the cell media was replaced with either supplemented Stempro Osteogenesis Differentiation Media (Thermo), Stempro Chondrogenesis Differentiation Media or Stempro Adipogenesis Differentiation Media. Cells were cultured for 21 days, with media changes every other day. Cells were fixed in 4% PFA for 30 minutes and stained accordingly to establish protocols [37] Osteogenic differentiation was shown by formation of calcium-hydroxyapatite-positive areas (Alizarin Red staining). Chondrogenic differentiation was assessed with toluidine blue staining to demonstrate extracellular matrix mucopolysaccharides. Confirmation of adipogenic differentiation by intracellular accumulation of lipid-rich vacuoles stainable with oil red O (Sigma). Cells were stained with 0.16% oil red O for 20 minutes.

**2.10 Lytic CYP3A4 activity assay**

CYP3A4 activity was measured on protein extracts from days 6 of LOs maturation following manufacture`s instructions (Promega). Data was normalized to protein concentration.

**2.11 CDFDA uptake**

CDFDA (Sigma) was added to a final concentration of 10 μM to the cell media and incubated for 30 min in 37°C. LOs were washed twice with DPBS and cell media was refilled. Cellular uptake and transportation was evaluated in a fluorescence microscope (Nikon).

**2.12 Proteomics**

Proteomics was performed in LO at day 12. For secretome analysis, paired combinations of endothelial cells and MSCs were plated at equal ratios and at high confluence (80%). Cells were culture for 24h using 50/50 mixture of endothelial/MSC, washed 3X with Dulbecco modified PBS and cultured for 48h in serum-free high glucose DMEM. Media was collected, filtered through a 20 μm membrane and concentrated using 3 kDa filters (Millipore). Briefly, for protein extraction and digestion, samples were treated with 8M urea, followed by protein reduction with dithiothreitol (5mM for 25 min at 56 °C) and alkylation with iodoacetamide (14 mM for 30 min at room temperature). Urea was diluted to a final concentration of 1.6M with 50 mM ammonium bicarbonate, and 1 mM of calcium chloride was added to the samples for trypsin digestion for 16h at 37°C (2 μg of trypsin). The reaction was stopped with 0.4% formic acid, and peptides were desalted with C18 stage tips, dried in a vacuum concentrator, reconstituted in 0.1% formic acid and stored at −20 °C for subsequent analysis by LC-MS/MS. The peptide mixture (total volume of 4.5 μL) was analyzed using an LTQ Orbitrap Velos (Thermo Fisher Scientific) mass spectrometer coupled to nanoflow liquid chromatography on an EASY-nLC system (Proxeon Biosystems) with a Proxeon nanoelectrospray ion source. Peptides were subsequently separated in a 2–90% acetonitrile gradient in 0.1% formic acid using a PicoFrit analytical column (20 cm × ID75, 5 μm particle size, New Objective) at a flow rate of 300 nL/min over 212 min, in which a gradient of 35% acetonitrile is reached in 175 min. The nanoelectrospray voltage was set to 2.2 kV, and the source temperature was set to 275 °C. The instrument methods employed for LTQ Orbitrap Velos were set up in DDA mode. Full scan MS spectra (m/z 300–1600) were acquired in the Orbitrap analyzer after accumulation to a target value of 1e6. Resolution in the Orbitrap was set to r = 60,000, and the 20 most intense peptide ions (top 20) with charge states ≥2 were sequentially isolated to a target value of 5000 and fragmented in the high-pressure linear ion trap by CID (collisioninduced dissociation) with a normalized collision energy of 35%. Dynamic exclusion was enabled with an exclusion size list of 500 peptides, an exclusion duration of 60 s and a repetition count of 1. An activation Q of 0.25 and an activation time of 10 ms were used.

Twelve LC-MS/MS runs were performed. Raw data were processed using MaxQuant v1.3.0.3 software, and MS/MS spectra were searched against The Human UniProt database (released January 7, 2015, 89,649 sequences, and 35,609,686 residues) using the Andromeda search engine. As search parameters, a tolerance of 6 ppm was considered for precursor ions (MS search) and 0.5 Da for fragment ions (MS/MS search), with a maximum of two missed cleavages. A maximum of a 1% false discovery rate (FDR) was set for both the protein and peptide identification. Protein quantification was performed using the LFQ algorithm implemented in MaxQuant software, with a minimal ratio count of 2 and a window of 2 min for matching between runs. Statistical analysis was performed with Perseus v1.2.7.4 software, which is available in the MaxQuant package. Identified protein entries were processed, excluding reverse sequences and those identified “only by site” entries. Contaminants were not removed from the dataset because serum albumin is of interest in the study of LO. Protein abundance, which was calculated based on the normalized spectrum intensity (LFQ intensity), was log2-transformed, and the dataset was filtered by minimum valid values in at least one group. Missing values for the LFQ intensity were not imputed as random numbers, the mean and standard deviation of which were selected to best simulate low abundance values close to the noise level (imputation width = 0.3, shift = 1.8).

Significance was assessed by comparing all conditions and samples using ANOVA and Student’s t test to identify differentially expressed proteins between specific groups (P value < 0.05). Exclusive and common proteins from each comparison are presented as a Venn diagram generated using the InteractiVenn tool. For data visualization, heat maps with z-score values of log2 LFQ intensities.

- 1. **Data availability**

The mass spectrometry proteomics data have been deposited to the ProteomeXchange Consortium via the PRIDE partner repository with the dataset identifier PDX013190

- 1. **Ethics statement**

The experimental procedures involving samples from human subjects were approved by the Ethics Committee of Instituto de Biociências at Universidade de São Paulo, Brazil (Protocol number 1.294.118).

**3. Supplementary Figures Caption**

**Supplementary Figure 1 – Cellular characterization**

(A) Representative phase contrast images of iPS cell lines in culture (bar = 200 um). (B) Representative data from F9048 iPS characterization. RT-qPCR for *PAX2*, *CXCR4*, *T*, *LIN28*, *NANOG* and *OCT3/4*. Also, flow-cytometry for OCT4 and SSEA4. (C) Representative flow cytometry panel of cell lines F8799 and F7405 stained for terminal differentiation markers of hepatocyte (ALB and UGT1A1), endothelial (VECAD and CD31) and MSC (CD90 and CD73). (D) HAEC cell line stained for CD31 and VECAD (bar = 50 μm) and dp-MSC cell line stained for CD105 (bar = 50 μm). (E) Representative data from F9048 RT-qPCR of iPS-derived endothelial cells for *NOTCH4*, *PDPN* and *EPHB4* genes. (F) A1AT and Urea concentration in media supernatant of cultured LO at day 6 and day12. (G) Lytic CYP3A4 activity assay normalized to protein concentration (n = 3, biological replicates; one-way Anova with Tukeys` post-hoc, * = p < 0.05, ** = p < 0.01, *** = p < 0.01). (H) Representative YAP/TAZ western blotting staining and analysis (n = 3, biological replicates; one-way Anova with Tukeys` post-hoc). (I) Representative IF tissue staining for CK18/CD31, CD105/VECAD and MRP1. Representative CDFDA fluorescent staining.

**4. Supplementary References**

10. Miller, E., Kobayashi, G., Musso, C., Allen, M., Ishiy, F., de Caires, L., Goulart, E., Griesi-Oliveira, K., Zechi-Ceide, R., and Richieri-Costa, A. et al. EIF4A3 deficient human iPSs and mouse models demonstrate neural crest defects that underlie Richieri-Costa-Pereira syndrome. Human Molecular Genetics. 2017;26:2177-91.

33. Caires-Júnior, L., Goulart, E., Melo, U., Araujo, B., Alvizi, L., Soares-Schanoski, A., de Oliveira, D., Kobayashi, G., Griesi-Oliveira, K., and Musso, C. et al. Discordant congenital Zika syndrome twins show differential in vitro viral susceptibility of neural progenitor cells. Nature Communications. 2018;9.

34. Cameron K, Tan R, Schmidt-Heck W, Campos G, Lyall MJ, Wang Y, Lucendo-Villarin B, Szkolnicka D, Bates N, Kimber SJ, Hengstler JG, Godoy P, Forbes SJ, Hay DC. Recombinant Laminins Drive the Differentiation and Self-Organization of hESC-Derived Hepatocytes. Stem Cell Reports. 2015;5(6):1250-62.

35. Sriram, G., Tan, J., Islam, I., Rufaihah, A., and Cao, T. Efficient differentiation of human embryonic stem cells to arterial and venous endothelial cells under feeder- and serum-free conditions. Stem Cell Research & Therapy. 2015;6.

36. Ishiy, F., Fanganiello, R., Griesi-Oliveira, K., Suzuki, A., Kobayashi, G., Morales, A., Capelo, L., and Passos-Bueno, M. Improvement of In Vitro Osteogenic Potential through Differentiation of Induced Pluripotent Stem Cells from Human Exfoliated Dental Tissue towards Mesenchymal-Like Stem Cells. Stem Cells International. 2015;2015:1-9.

# 37. Jazedje T, Bueno D, Almada B, Caetano H, Czeresnia C, Perin P, Halpern S, Maluf M, Evangelista L, Nisenbaum M, Martins M, Passos-Bueno M and Zatz M. Human fallopian tube mesenchymal stromal cells enhance bone regeneration in a xenotransplanted model. Stem Cell Rev. 2012;8(2):355-6

**Supplementary Figure**


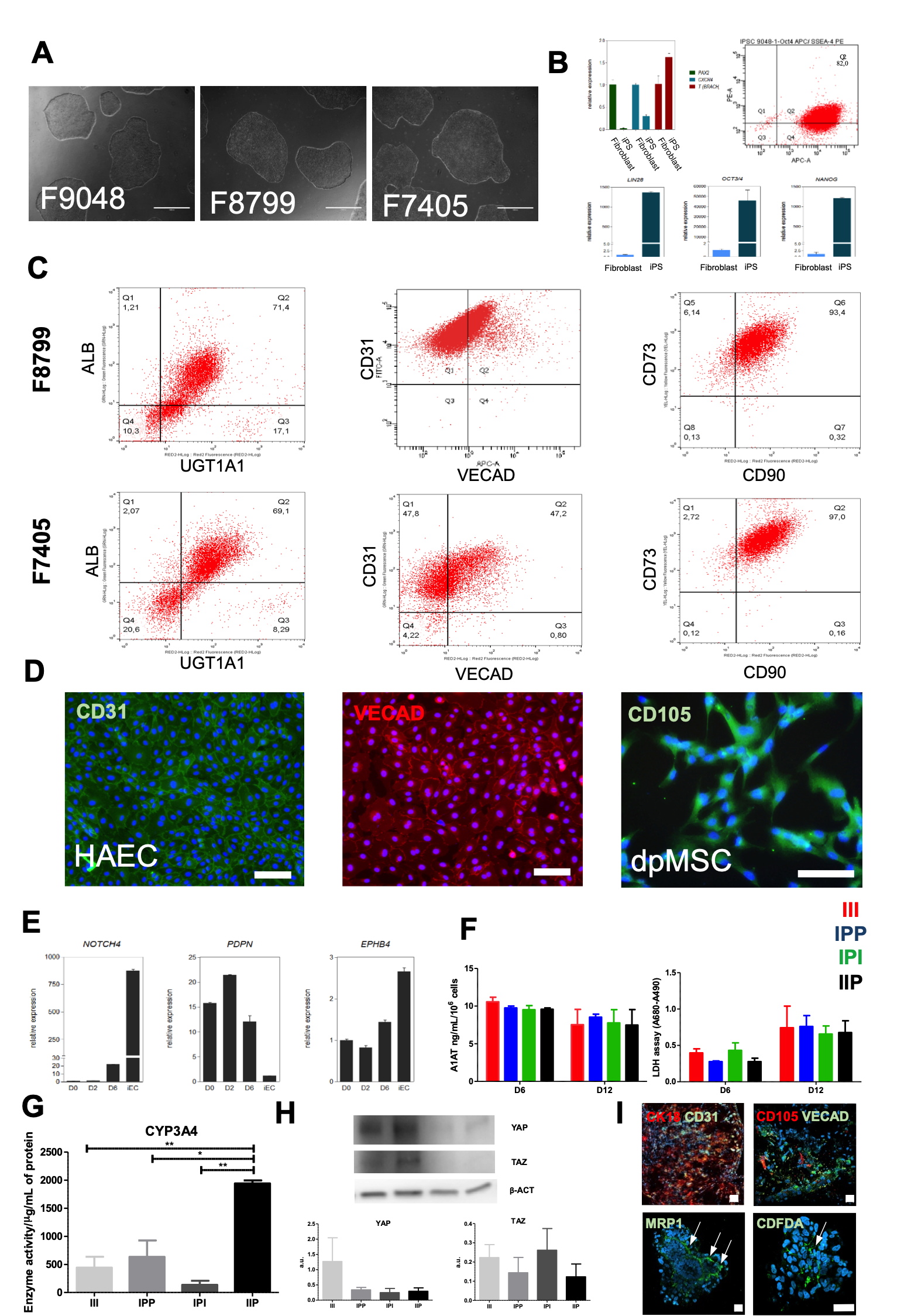

Supplement: Supplementary file 1 — Supplementary tables, methods, and figures (DOCX 1729 kb) [file 13287_2019_1367_MOESM1_ESM.docx]
